# Effects of Foliar Application of Copper and Gold Nanoparticles on *Petroselinum crispum* (Mill.)

**DOI:** 10.3390/nano15040280

**Published:** 2025-02-12

**Authors:** Alexandra Peshkova, Inga Zinicovscaia, Ludmila Rudi, Tatiana Chiriac, Nikita Yushin, Liliana Cepoi

**Affiliations:** 1 Joint Institute for Nuclear Research, 6 Joliot-Curie Str., 141980 Dubna, Russia; ynik_62@mail.ru; 2 Doctoral School of Natural Sciences, Moldova State University, M. Kogalniceanu Str., 75A, MD-2009 Chisinau, Moldova; 3 Horia Hulubei National Institute for R&D in Physics and Nuclear Engineering, 30 Reactorului Str., 077125 Măgurele, Romania; 4 Institute of Microbiology and Biotechnology, Technical University of Moldova, 1 Academiei Str., MD-2028 Chisinau, Moldova; ludmila.rudi@imb.utm.md (L.R.); tatiana.chiriac@imb.utm.md (T.C.); liliana.cepoi@imb.utm.md (L.C.)

**Keywords:** metal nanoparticles, parsley, nanoparticle accumulation, biochemistry, antioxidant activity, translocation factor, risk assessment

## Abstract

The unintentional release of nanoparticles in the atmosphere and their targeted application to improve plant productivity requires detailed study. The translocation features of copper and gold nanoparticles applied by spraying in the concentration range of 1–100 mg/L in *Petroselinum crispum* (Mill.) tissues during a 10-day experiment were investigated. Atomic absorption spectrometry and inductively coupled plasma atomic emission spectroscopy showed that copper and gold nanoparticles applied to the leaves’ surface could accumulate in plant organs. A dose-dependent increase in the content of copper and gold in the aerial parts of parsley was revealed. The content of copper in leaves treated with nanoparticles was 1–2.3 times higher than the control, while the content of gold exceeded control values 2–116 times. The effect of nanoparticles on plants’ biochemical composition was assessed. The antioxidant tests showed an ambiguous response at exposure to metal nanoparticles. Copper nanoparticles at the applied concentration consistently reduced both chlorophyll and carotenoid content. Gold nanoparticles enhanced the chlorophyll and carotenoid level at low concentrations (1 mg/L) and significantly inhibited it at higher concentrations. The parsley exposed to nano-copper remained safe for human consumption, but parsley containing more than 14.9 mg/kg of gold may adversely affect human health.

## 1. Introduction

The release of nanoparticles into the environment occurs both due natural processes, such as volcanic eruptions, forest fires, and rock weathering, and as a result of industrial use. Currently, the Nanotechnology Products Database lists more than 11,000 products containing nanoparticles (NPs) [1,2]. Metallic NPs based on gold, copper, silver, platinum, and zinc are used to solve a range of problems in medicine, including cancer diagnostics, the detection of pathogenic microorganisms, and the application as antimicrobial and antioxidant agents [3]. Gold NPs are widely applied for the targeted delivery of various vaccines, pharmaceuticals into the human body [4,5], and as cosmetics components [6]. Copper NPs are applied in building construction, increasing materials’ corrosion resistance [7]; the production of composites and super-strong materials [8]; in medicine as antimicrobial and bactericidal agents [9]; as additives in lubricants, polymers, for metal coatings, and inks; and in the agricultural sector [10,11,12,13]. The intensive production and use of NPs results in their release in large quantities into the environment. Thus, Keller A. et al. [13] noted that most copper compounds are released into the soil and sediments, where they accumulate to potentially toxic levels (>50–500 µg/L). The toxicity of copper compounds is generally ranked as follows: Cu^+2^ > nano Cu (0) > nano Cu (OH)_2_ > nano CuO > micron-scale Cu compounds. Au-containing particles of various shapes, up to 50 nm in size, were detected in wild *Erigeron canadensis* and *Boehmeria nivea*, collected in Guangdong Province, China [14]. The presence of NPs in the atmosphere leads to the deposition of nano dust on various surfaces, including plant leaves, soil surfaces, and water bodies [15,16,17].

At the same time, the use of metal NPs as fertilizers, growth enhancers, and pesticides is becoming increasingly common due to their special physical and chemical characteristics, high absorption, and surface and adhesive effects compared to larger materials. It has been shown that the use of non-toxic concentrations of NPs can promote seed germination and improve plant growth and yield [18,19]. Different modified NPs are introduced in agricultural soils and irrigation water to improve plants’ nutrient delivery and stress tolerance [20]. A number of studies have reported the effectiveness of using NPs under drought conditions [21,22,23,24,25]. Yasmeen et al. [22] showed that Fe and Cu NPs significantly improved spike length, grain/spike, and grain weight of wheat plants. Van Nguyen et al. [23] reported that the application of nano-CuO increased the carotenoid content in maize plants, thereby improving the efficiency of photosynthetic performance. The CuNPs foliar treatment enhanced salt stress tolerance by activating tomato plant antioxidant mechanisms [24]. NPs can be used as nanocapsules to transport and release herbicides, fertilizers, or genes to specific target areas of plants to achieve the desired effects [25].

It should be mentioned that the uptake, transport, and accumulation of NPs are influenced by many factors such as humidity and temperature, NP properties (size and shape), and plant physiological characteristics [26,27,28]. Venzhik et al. [29] observed an increase in the frost resistance of winter wheat after seed treatment with nanogold. Malandrakis et al. [30] demonstrated the ability of CuNPs, ZnONPs, and AgNPs to inhibit the growth of fungal strains depending on the dose applied. The application of nanofertilizers can be carried out in two ways, by irrigation and surface application to foliage [31,32,33,34,35]. The foliar application of nutrients increases the efficiency of fertilizers compared to traditional soil–root irrigation. The spraying of gold nanoparticles on *Brassica juncea* leaves had a stimulating effect on plants in a field experiment, with a positive effect on plant height, stem diameter, number of branches, and yield [36]. López-Vargas et al. [37] revealed a positive effect of the foliar treatment of copper NPs on the tomato fruit (*Solanum lycopersicum* L.) quality and the contents of the antioxidant compounds.

Despite the positive effects on plants, the long-term use of nanoparticle-based products may pose a risk of accumulation in soil and for the ecosystem as a whole. Zhao et al. [38] identified that bacterial communities were altered after exposure to CuO nanoparticles. The release of Cu ions due to the high bioavailability and dissolution of CuONPs in soil was the major reason for NP toxicity to microorganisms. Xiong et al. [39] observed that the biotransformation of CuNPs in plant leaves pose a potential human health risk. The foliar application of nano-CuO (10 and 250 mg/plant) had a negative effect on lettuce and cabbage plants’ biomass and photosynthetic activity, expressed as necrosis, chlorosis, and growth delay [39]. The phytotoxic effect of AuNPs, expressed as a significant decrease in the chlorophyll *a* content compared to the control, was detected after the foliar treatment of wheat with NPs in concentrations of 20 and 30 mg/L [40]. The penetration of engineered NPs into plant segments, accumulation in tissues, and further participation in trophic transfer creates risks for higher-ranking consumers [13,41,42]. Thus, NPs are bioavailable in various ecosystems and can be transferred from one organism to another in trophic networks. Plants have been reported to uptake gold NPs and transfer them to hornworms [43]. The greens contaminated with NPs can negatively affect human health, so it is very important to assess NP uptake by edible plants. To our knowledge, this is the first study investigating the impact of the foliar treatment of *Petroselinum crispum* (Mill.) with copper and gold NPs.

The main goal of this work was (1) to assess the uptake of gold and copper NPs in *Petroselinum crispum* (Mill.) segments under foliar spraying conditions, (2) to determine the effect of different concentrations of CuNP and AuNP solutions on the biochemical parameters and antioxidant activity of plants, and (3) to assess the risk to human health associated with the consumption of contaminated plants.

## 2. Materials and Methods

### 2.1. Materials

PVP-coated copper and gold NP solutions (200 mg/L by metal) were acquired from the M9 company (Tolyatti, Russia). In the laboratory, the initial solution was diluted to obtain working solutions with NP concentrations of 1, 5, 10, 50, and 100 mg/L. Soil of the brand Terra Vita (Nevatorf company, Moscow, Russia) was chosen, as it is actively used for the year-round cultivation of vegetables in greenhouses. The soil pH was 6–6.5, and it contained high-moor peat, biohumus, alluvial sand, agroperlite, limestone flour, and macro- and microelements [44]. *Petroselinum crispum* (Mill.) seeds were acquired from the Poisk agricultural company (Moscow region, Russia). Parsley was chosen as the object of study due to its active use in the food industry, as well as for pharmaceutical purposes [45]. Seeds were planted in the 500 mL pots filled with soil and for two months were watered with tap water. Once the plants reached a height of 10 cm, they were used for the experiment.

### 2.2. Experimental Design

To assess the effect of NPs, copper and gold NPs were applied to the formed adult plants using a polypropylene sprayer every 2 days for 10 days. During one treatment, each plant was sprayed with 2 mL of NP solution. To prevent solution runoff from the leaf surface, soil was shielded with thick paper to prevent contamination. Control plants were not treated with nanoparticle solutions. All experiments were conducted in three repetitions.

The temperature in the laboratory was maintained at 20–21 °C. After 10 days of the experiment, soil samples were taken and each plant was divided into the arial part and roots. Plants were cleaned with deionized water to remove the NP solution and soil residues. The first stage of sample drying took place at a temperature of 50 °C; the second stage of homogenized sample drying was conducted out at 105 °C until constant weight. For the biochemical analyses, fresh leaf biomass was frozen at −80 °C.

### 2.3. Optical Emission and Atomic Absorption Spectrometry (ICP-OES and AAS)

The shape and size of NPs was described using a Thermo Scientific Talos F200i microscope (Waltham, MA, USA). The sample preparation was performed, similar to [46]. The content of copper in plant and soil samples was determined using a PlasmaQuant PQ 9000 Elite (Analytik Jena, Jena, Germany) spectrometer. More details about sample preparation and analysis can be found elsewhere [46]. A Thermo Scientific iCE 3000 (Waltham, MA, USA) electrothermal atomic absorption spectrometer was used to determine the gold content in samples. The gold ICP standard in HCl 7% 1000 mg/L was used to build the calibration curve. Measurements were carried out using an ELC-type cell. The temperature of pyrolysis was 800 °C, the atomization temperature was 2100 °C, and the gas flow was 0.2 L/min. The measurement of copper and gold concentrations in solutions by AAS and ICP-OES methods was performed in triplicate, and the results are presented as the average ± standard deviation.

### 2.4. Biochemical Analysis

#### 2.4.1. Preparation of Parsley Plant Extracts

The parsley plants were ground in a mortar with a 50% hydro-ethanolic solution (0.01 g plant material per 1 mL hydro-ethanolic solution) until a homogeneous suspension was formed. Extraction was conducted by stirring for 180 min. Next, the extract was separated from the biomass by centrifugation.

#### 2.4.2. Determination of Pigments (Total Chlorophyll and Carotenoids)

The pigment content was calculated based on molar extinction coefficients after measuring the absorbance of the extract at 450 nm and 665 nm, respectively, as described by Ritchie [47] and Rodriguez-Amaya [48].

#### 2.4.3. Antioxidant Activity

The antioxidant activity was evaluated using 2,2′-azino-bis 3-ethylbenzothiazoline-6-sulfonic acid, the (ABTS) radical cation method [49], and with 2,2-diphenyl-1-picrylhydrazyl (DPPH) [50]. More information about radical solution preparation can be found in [46,51]. The absorbance of the ABTS radical was measured at 734 nm and of DPPH at 515–517 nm. The antioxidant activity was calculated based on the reduction in radical solution extinction values (% inhibition).

### 2.5. Data Evaluation

The calculation of the translocation factor (*TF*), demonstrating the ability of plants to move metals from the above-ground part to the root system, was calculated using Equation (1):

(1) $$TF=\frac{C_{R}}{C_{L}}$$

where *C_R_*—metal content in roots; *C_L_*—metal content in leaves.

The estimated daily intake (*EDI*) and the hazard quotient (*HQ*) were calculated using Equations (2) and (3):

(2) $$EDI=\frac{C_{i}\;*\;IR\;*\;EF\;*\;ED}{AT\;*\;BW}$$

(3) $$HQ=\frac{EDI}{RfD}$$

where *C_i_*—metal content in the aerial parts of parsley, mg/kg; *IR*—green consumption rate, kg; *EF*—exposure frequency, 365 days/year; *ED*—duration of exposure, 70 years; *AT*—average exposure time, *EF* × *ED*; and *BW*—average body weight, 70 kg. *RfD*—daily oral reference dose—for copper, the value *RfD* 0.04 mg·kg/day is used [52]. To calculate the *HQ* for gold, the *RfD* was taken as 1.32 µg kg/day, based on the use of a nutritional supplement E175 [51,52,53].

## 3. Results and Discussion

### 3.1. Nanoparticle Characterization

**Figure 1:**
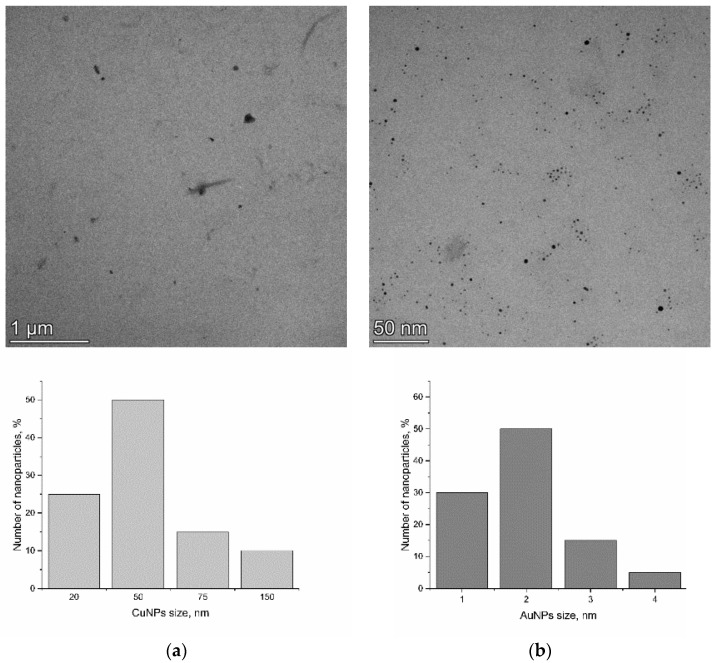
TEM images of CuNPs (**a**) and AuNPs (**b**) and their size distribution.

According to TEM, both types of NPs were of spherical shape. Most of the CuNPs had sizes in the range of 20–50 nm and formed aggregates with a size up to 150 nm. AuNPs with particle diameters of 1–4 nm were distributed uniformly in the solution (Figure 1).

### 3.2. Copper and Gold Distribution in Petroselinum crispum (Mill.) Segments

**Figure 2:**
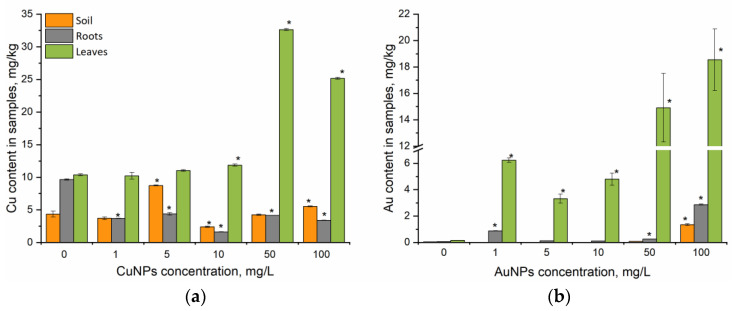
Copper (**a**) and gold (**b**) content in soil and parsley segments under foliar spray conditions during a 10-day experiment (* *p* < 0.05 for differences between control and experimental samples; the error bars represent the standard deviation of the measurements).

The content of copper accumulated in soil and parsley leaves and roots after the experience of plants being spraying with NPs can be seen in Figure 2a.

The levels of copper in control soil, root, and leaf samples of parsley were 4.37, 9.65, and 10.38 mg/kg in terms of dry weight, respectively (Figure 1a). When spraying plants with CuNPs in concentrations of 1, 5, and 10 mg/L, the content of copper in the leaves corresponded to its content in control plants. CuNPs concentrations of 50–100 mg/L resulted in an increase of copper content in the leaves 3 and 2.5 times, respectively, compared to the control. At the same time, a reduction in copper content in the roots of plants exposed to NPs was observed at all applied concentrations (2.2–6 times). The correlation coefficient between the copper content in leaves and roots was r = 0.28 at *p* < 0.005. The maximum decrease in the copper content in roots (up to 1.62 mg/kg, which was six times lower than the control) was observed at plants sprayed with 10 mg/L of CuNPs.

The gold content in the control soil samples, roots, and aerial parts of parsley corresponded to 0.046, 0.07, and 0.16 mg/kg, respectively. The gold content in the green parts of the experimental parsley increased dose-dependently from 6.23 to 18.5 (at *p* < 0.005 r = 0.95), which was 21–115 times higher than the control (Figure 2b). The less pronounced pattern (at *p* <0.005; r = 0.81) was observed for the root system (from 0.88 to 2.87 mg/kg). In this case, gold content in experimental plants treated with 100 mg/L of AuNPs was 42 times higher than in control samples. When the leaf surface was exposed to 1–10 mg/L of AuNPs, the gold content in the soil was below the technique detection limit; however, at a AuNP concentration of 100 mg/L, it increased 30 times compared to the control (0.046 mg/kg).

### 3.3. Translocation Factor

At all applied CuNP and AuNP concentrations, the translocation coefficient *TF* < 1, which indicates low element transport from the parsley aerial part to the root system (Table 1).

### 3.4. Effect of AuNPs and CuNPs on Parsley Biochemical Parameters

**Figure 3:**
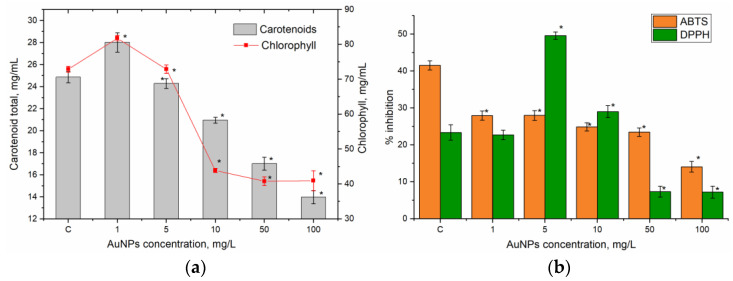
Effect of different concentrations of AuNPs on pigment content and antioxidant activity in parsley (* *p* < 0.0001 for differences between control and experimental samples; the error bars represent the standard deviation of the measurements).

The effects of different concentrations of AuNPs on pigment content and antioxidant activity in parsley extracts are summarized in Figure 3. The impact of NPs on pigment content in parsley varied according to the applied concentration. At 1 mg/L, there was a significant increase in carotenoids (12.68%, *p* = 0.0029) and total chlorophyll content (by 12.21%, *p* < 0.0001) relative to the control. At a AuNP concentration of 5 mg/L, levels of carotenoids and chlorophyll were similar to those in the control, but higher NP concentrations resulted in a marked reduction in their content. Specifically, at 10 mg/L, carotenoids decreased by 15.7% (*p* < 0.0001), and at 50 mg/L, they were reduced by 31.6% (*p* < 0.0001). At 100 and 200 mg/L, carotenoid levels dropped by 43.5–43.8% compared to the control (*p* < 0.0001). Total chlorophyll content decreased independently in a range of 10–100 mg/L, with reductions of 39.8–43.8% relative to the control (Figure 3a).

The antioxidant activity of parsley extracts treated with various AuNP concentrations also showed notable changes (Figure 3b). The ABTS assay, which measures antiradical activity via electron transfer, showed a reduction in antioxidant capacity by over 32% in plants treated with 1 and 5 mg/L of AuNPs compared to the control. As concentration increased, ABTS activity declined, reaching a reduction of more than 60% at 100 mg/L. Conversely, the DPPH assay, which assesses proton transfer-based activity, showed that antiradical activity against DPPH remained stable or even increased at a AuNP concentration of 1–10 mg/L (an increase of approximately 100% at 5 mg/L and of 24% at 10 mg/L). However, at higher AuNP concentrations, DPPH activity decreased by 68.4–70.2% compared to the control.

**Figure 4:**
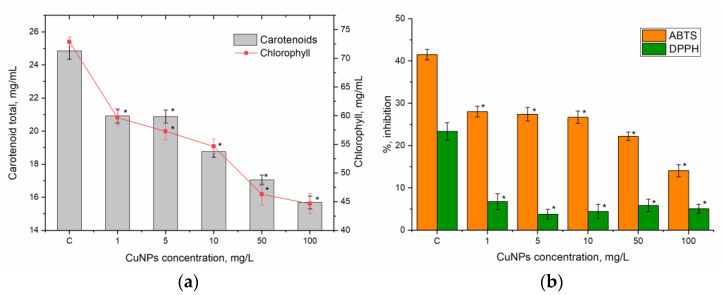
Effect of different concentrations of CuNPs on pigment content and antioxidant activity in parsley (* *p* < 0.001 for differences between control and experimental samples; the error bars represent the standard deviation of the measurements).

The results regarding CuNPs’ effect on parsley are shown in Figure 4. In contrast to AuNPs, CuNPs generally had a negative effect on pigment levels, even at low concentrations (Figure 4a). At 1 mg/L, carotenoids decreased by 15.9% and total chlorophyll by 18.2%. The reduction intensified with higher concentrations; at 100 mg/L, carotenoid levels dropped by 44.4% and chlorophyll by 47.0% compared to the control. This dose-dependent effect exhibited a high correlation (correlation coefficients > 0.9).

Regarding antioxidant activity, the ABTS and DPPH assays indicated lower activity in parsley extracts treated with CuNPs (Figure 4b). The ABTS inhibition capacity was 32.4–35.7% lower in plants treated with CuNPs in a concentration range of 1–10 mg/L, while a concentration of 100 mg/L resulted in a reduction of 66%. DPPH activity was consistently 71–84% for all CuNPs without significant variation among them.

### 3.5. Human Health Risks of Consuming Potentially Contaminated Parsley

Based on the WHO data on the desirable consumption of at least 400 g of vegetables per day and the recommendation that one serving in five should consist of green leafy vegetables [54,55], the value of 80 g of fresh greens per day was used to calculate the EDI and HQ. When converted to dry weight, a factor of 0.12 was used for greens [56]. The EDI and HQ values are presented in Table 2.

The obtained results indicate an increase in the EDI values with an increase in the gold and copper content in the edible parts of parsley. HQ < 1 was obtained for all CuNP concentrations and for AuNP concentrations of 1–10 mg/L, indicating a low risk for human health. At the same time, it needs to be mentioned that for parsley treated with 50–100 mg/L of AuNPs, HQ exceeded a unit, pointing at possible negative consequences for human health.

## 4. Discussion

The effect of NPs on plants is dependent on many factors, including dosage, the particle deposition frequency, plant species, foliar microorganisms, particle size, and the shape of NPs [27,51,57,58,59]. The NP dosage is one of the parameters that has a great influence on their toxicity for plants. As soon as the amount of applied copper exceeds a critical value, it becomes toxic to plants, leading to stunted growth and the chlorosis of leaves [49]. The toxic effects of Cu on plants are also associated with the formation of OH° radicals, which cause the alteration and disruption of macromolecular metabolic pathways and can lead to genotoxic effects in exposed leaves [59]. Leaf spraying with CuNPs and AuNPs did not cause visual damage of parsley leaves during a 10-day experiment (Figure S1).

The application of CuNPs resulted in their accumulation in the green parts of *Petroselinum crispum* (Mill.) and demonstrated weak translocation into the root system, which is consistent with other studies. Thus, Kohatsu et al. [60] reported that copper, both as CuSO_4_ and CuONPs, when sprayed onto lettuce leaves, was localized in the leaves and no translocation to the roots was observed. López-Luna [61] found greater uptake and translocation of CuNPs by avocado plants exposed to NPs through foliar sprays compared to root treatment and injections [61]. Plants can absorb NPs through their stomata, cracks or water pores, ion channels, protein carriers, endocytosis, stigma, wound, and trichomes. The foliar application of nano-sized copper may restrict root and shoot growth and had symptoms of stomatal closure [26]. Also, the localization of NPs in plants is influenced by the pore size of the cell wall, the fluid flow rate, and other factors [62]. It is known that cell walls and waxes act as physical barriers, preventing leaf cell necrosis and genotoxic effects caused by excessive metal particle deposition [26]. The formation of CuNP aggregates in the solution could be another reason of weak copper transport to the root system.

As for the treatment of plants with AuNPs, gold was mainly accumulated in leaves and to a lesser extent in roots and soil. The increase in gold content in the aerial parts of *Brassica juncea* after spraying with NPs was described by Arora et al. [36]. Ha et al. [63] assumed that ultrafine particulate matter is absorbed through stomatal pathways. The increase in gold content in roots and soil when treated with a highly concentrated solution of AuNPs at 100 mg/L may be associated with the transport of gold through plant tissues to the root system and its release into the rhizosphere part of the soil. Avellan et al. [64] also noted that most of the translocated AuNPs were accumulated in young wheat shoots (10−30%) and roots (10−25%), and 5−15% of NPs < 50 nm were released into the rhizosphere soil.

The application of NPs had a negative impact on plants, proved by the decrease in the level of pigments. If, in the case of AuNPs, the reduction in the level of carotenoids and total chlorophyll was observed at high NP concentrations (10–100 mg/L), CuNPs lowered pigment content at all applied concentrations. In previous studies, the effects on pigments similar with those observed for AuNPs at low-dose exposure to NPs has been shown. For instance, low doses of AgNPs have enhanced pigment levels in *Vigna radiata*, and chlorophyll content in waxy maize seeds treated with AuNPs increased by up to 53% depending on the NP concentration [65,66]. In *Brassica juncea*, a 29% increase in total chlorophyll was also demonstrated when exposed to a low dose of AuNPs [36]. The positive response to NP treatment at low concentrations may be associated with the hormesis effect, a favorable biological response to the exposure to low concentrations of toxins and other stressors [67]. Such an increase may reflect an initial boost in photosynthetic activity, which can also correlate with higher antioxidant activity. For example, in *Arabidopsis thaliana*, AuNPs provoked an increase in DPPH radical inhibition by over 3.5 times at 10 μg/mL [68].

CuNPs and AuNPs also affected the antioxidant activity of experimental parsley plants, mostly resulting in its decrease, especially at high NP concentrations. Studies on the effects of CuNPs on antioxidant activity in other plants reflect similar results. For instance, in rice seedlings (*Oryza sativa* L.), CuNP exposure led to a substantial reduction in chlorophyll and carotenoid content, especially at concentrations up to 250 mg/L [59]. A similar reduction in chlorophyll content was observed in cucumber (*Cucumis sativus*) at CuNP concentrations of 50, 100, and 200 mg/L [69] and in tomato leaves treated with 10, 50, and 250 mg/L of NPs [70]. These findings align with the obtained results, underscoring the phytotoxic potential of CuNPs across various plant species.

Since green parsley is widely consumed by population, EDI and HI values were calculated for plants sprayed with NP_S_. The EDI values proved to be directly proportional to copper or gold content in plants. AuNPs may present a hazard for human health at high concentrations. No health risk was observed during the consumption of spinach irrigated with water containing zinc oxide NPs [71]. Sutulienė and co-authors [72] have showed that HQ values did not exceed 1.0 when CuO and ZnO NPs were applied to treat ice plant (*Mesembryanthemum crystallinum* L.) leaves.

## 5. Conclusions

During the 10-day application of CuNP and AuNP solutions to the leaf surface of *Petroselinum crispum* (Mill.), differences in the accumulation and transfer of copper and gold were revealed. At all applied NP concentrations, copper content changed in the following order: leaves > soil > roots and that of gold was leaves > roots > soil. Compared to the control, copper content in the root system significantly decreased, while an increase of gold content was observed. AuNPs enhanced chlorophyll and carotenoid levels at low concentrations (1 mg/L) in parsley. However, higher AuNP concentrations resulted in significant reductions in pigment content, showing a threshold beyond which AuNPs may inhibit pigment synthesis. Conversely, CuNPs consistently reduced both chlorophyll and carotenoid content across all tested concentrations, suggesting greater phytotoxicity compared to AuNPs. A dose-independent suppressive effect of NPs on antioxidant response was revealed. Although copper content in the edible parts of parsley increased after spraying leaves with CuNPs, parsley remained safe for human consumption. However, calculations have shown that the consumption of parsley containing more than 14.91 mg/kg of gold can have an adverse influence on human health.

## Figures and Tables

**Table 1:** Copper and gold translocation factor (*TF*) in parsley tissues.

Concentration of NPs in Solution, mg/L	TF (Cu)	TF (Au)
Control	0.930 ± 0.09	0.420 ± 0.01
1	0.362 ± 0.02	0.141 ± 0.001
5	0.397 ± 0.02	0.038 ± 0.0002
10	0.136 ± 0.002	0.025 ± 0.0007
50	0.127 ± 0.001	0.018 ± 0.0006
100	0.135 ± 0.004	0.155 ± 0.003

**Table 2:** EDI and HQ values calculated for green parts of parsley treated with copper and gold NPs.

	Concentration of NPs Solutions, mg/L	Metal Content in Leaves, mg/kg	EDI	HQ
Cu	Control	10.38 ± 0.52	1.19 × 10^−3^	0.03
	1	10.23 ± 0.2	1.17 × 10^−3^	0.03
	5	11.04 ± 0.13	1.26 × 10^−3^	0.03
	10	11.86 ± 0.15	1.36 × 10^−3^	0.03
	50	32.65 ± 0.16	3.73 × 10^−3^	0.09
	100	25.17 ± 0.15	2.88 × 10^−3^	0.07
Au	Control	0.16 ± 0.007	1.84 × 10^−5^	0.01
	1	6.23 ± 0.16	7.13 × 10^−4^	0.54
	5	3.33 ± 0.37	3.80 × 10^−4^	0.29
	10	4.80 ± 0.46	5.48 × 10^−4^	0.42
	50	14.91 ± 2.6	1.70 × 10^−3^	1.29
	100	18.55 ± 2.3	2.12 × 10^−3^	1.61

## Data Availability

Data are contained within the article.

## Supplementary Materials

The following supporting information can be downloaded at: https://www.mdpi.com/article/10.3390/nano15040280/s1, Figure S1: Photos of *Petroselinum crispum* (Mill.) (a) CuNPs, (b) AuNPs, and (c) control.
