# Supplementary figures and images for: Effects of Foliar Application of Copper and Gold Nanoparticles on Petroselinum crispum (Mill.)
Source: Nanomaterials (Basel). 2025 Feb 12;15(4):280. doi: 10.3390/nano15040280 (PMC11858691; doi:10.3390/nano15040280)

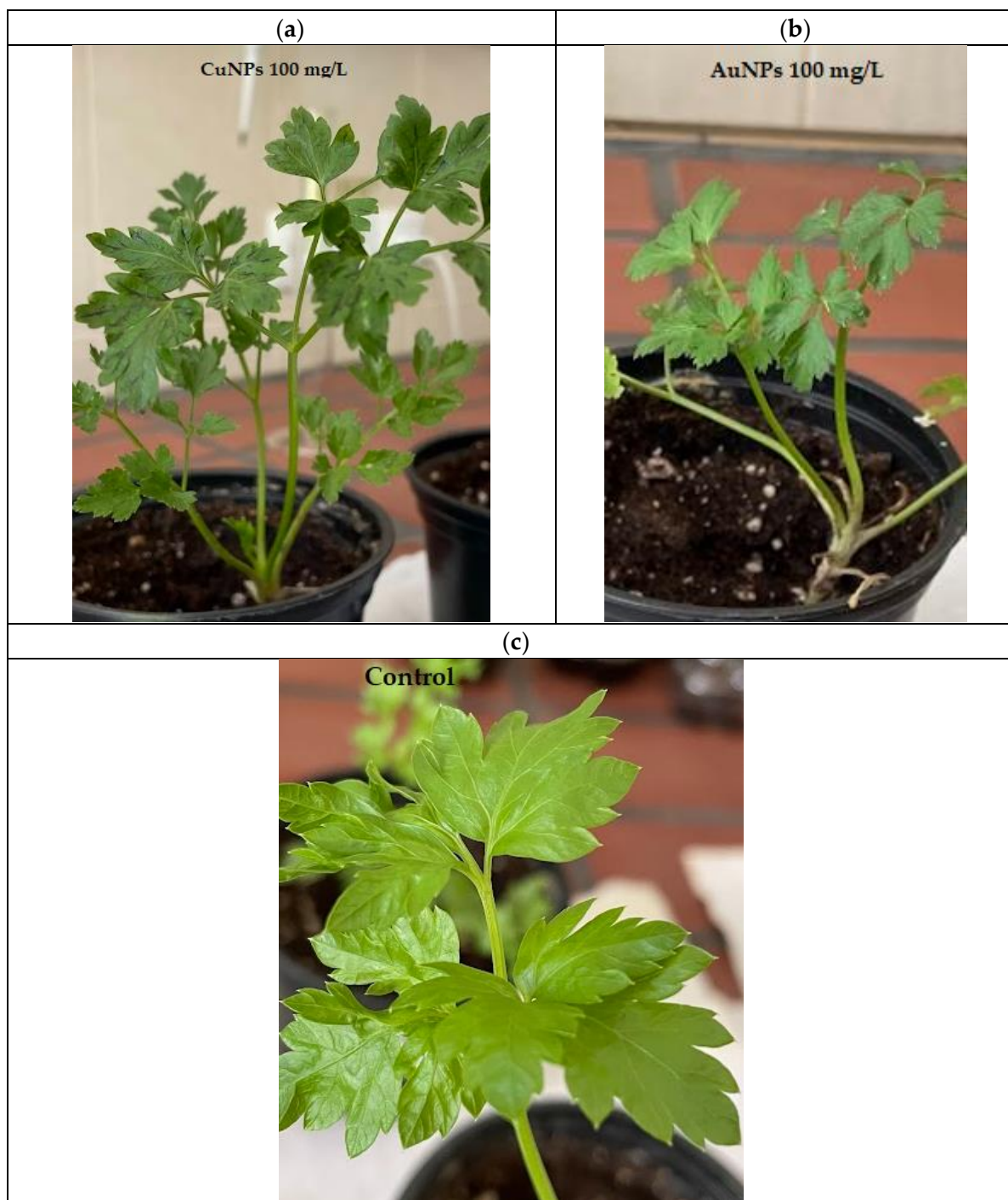

**Figure S1.** Photos of *Petroselinum crispum* (Mill.) (a) CuNPs, (b) AuNPs and (c) control.

Supplement: Supplementary file 1 [file nanomaterials-15-00280-s001.zip › nanomaterials-3456301-supplementary.pdf]
